# Microearthquakes preceding a M4.2 Earthquake Offshore Istanbul

**DOI:** 10.1038/s41598-018-34563-9

**Published:** 2018-11-01

**Authors:** Peter E. Malin, Marco Bohnhoff, Felix Blümle, Georg Dresen, Patricia Martínez-Garzón, Murat Nurlu, Ulubey Ceken, Filiz Tuba Kadirioglu, Recai Feyiz Kartal, Tugbay Kilic, Kenan Yanik

**Affiliations:** 1 Helmholtz-Centre Potsdam German Centre for Geosciences GFZ, Section 4.2 Geomechanics and Rheology, Telegrafenberg, 14473 Potsdam, Germany; 2 Freie Universität Berlin, Department of Earth Sciences, Malteser Strasse 74-100, 12249 Berlin, Germany; 3 AFAD Disaster and Emergency Management Presidency, Earthquake Department Ankara, 06510 Ankara, Turkey

## Abstract

A primary hurdle in observing small foreshocks is the detection-limit of most seismic networks, which is typically about magnitude M1-1.5. We show that a start-up test of a borehole-based seismic network with a much lower detection limit overcame this problem for an M_w_4.2 earthquake. This earthquake occurred offshore of Istanbul, Turkey, on a fault system that is likely to rupture in an M > 7 event in the coming decades. In the three days before and two after, a total of 62 or more earthquakes, including at least 18 foreshocks, came from the mainshock source area. The signal similarity of the foreshocks shows a clear increase during the hours before the M_w_4.2 mainshock. Similar foreshock sequences have recently been reported for a few well monitored M > 7 plate-boundary earthquakes. The sequence surrounding the M_w_4.2 gives the impression of stochastic failures that ended up interactively unloading stress concentrations. The M_w_4.2 mainshock then resulted from the accumulated release of significantly smaller events, as suggested by other field and laboratory studies.

## Introduction

A persistent question in seismology has been whether hazardous earthquakes show any kind of characteristic preparation process prior to their rupture initiation^1^. A plethora of laboratory rock deformation experiments, for example, are known to show sequences of much smaller failures preceding a through-going rupture^2,3^. These laboratory tests also showed that shortly before failure the spatial correlation of small failures increased significantly^4^. In the past, such small-scale failure processes have not been commonly observed for natural earthquakes along plate boundaries. Two reasons appear to be that (1) network detection thresholds were not low enough for seeing foreshocks of moderate-size events, and (2) due to the long recurrence times of large earthquakes, few such events have occurred within highly monitored areas.

Due to the densification of surface-based seismic networks^5–7^ and novel signal analysis techniques, observation of microearthquake seismicity preceding moderate to large earthquakes has recently improved. Specifically, subduction-zone megathrusts have been found to show this type of phenomena. Two examples are the 2011 M_w_9 Tohoku-Oki/Japan earthquake, with substantial pre-shock activity^6^, and the 2014 M_w_8.1 Iquique/Chile event, with systematic pre-seismic changes in magnitude-frequency relations^7^.

Correspondingly, it has also become possible to record much smaller microearthquakes before moderate-to-larger earthquakes along plate-bounding transform faults. These include the 1999 Izmit/Turkey M_w_7.4 earthquake^5,8^ and earthquakes along oceanic transform faults^9^. Likewise, corresponding activity has been found in studies of deep mine seismicity^10^.

It appears that foreshocks are at least two to three magnitudes lower than the mainshock they preceed^1–3,7,11^. Consequently, to observe the full foreshock processes, one needs to substantially lower magnitude detection limits in seismically active regions. One way to accomplish this is to instrument relevant regions using borehole sensors, as was done on the Parkfield segment of the San Andreas Fault by installing the Parkfield High Resolution Seismic Network. Such installations can greatly reduce seismic noise, thereby increasing earthquake signal-to-noise ratios^12^.

Here, we report on a 61-event microearthquake sequence surrounding an M_w_4.2 earthquake. The sequence was observed during a fortuitous 5-day startup test of the TESV borehole seismic station. These earthquakes occurred along the offshore Marmara section of the North Anatolian Fault Zone (NAFZ) in northwestern Turkey. At this site an M > 7 event is both overdue and not far from the 15-million-person population center of Istanbul.

The TESV station is part of the 7-station Geophysical Observatory at the North Anatolian Fault zone (GONAF). These borehole seismic stations surround the eastern Sea of Marmara^13^. Each of the GONAF sites include vertical and 3-C seismic sensors distributed at ~75 m intervals along 300-m deep boreholes. Consequently, they function as an array with locally very low magnitude-detection threshold - down to M~0. As a network for regional low-noise monitoring of the eastern Marmara target area it allows for accurate hypocenter locations down to M~1.

Cross-correlating TESV waveforms of all 62 events suggests spatial clustering of the entire sequence within an area of 1 km² - about the source size of the M_w_4.2 mainshock. Calculating running averages of the cross-correlation coefficients shows a well-defined increase during the hours before the mainshock – reminiscent of the lab results referred to above.

## Study Region and Data Base

**Figure 1:**
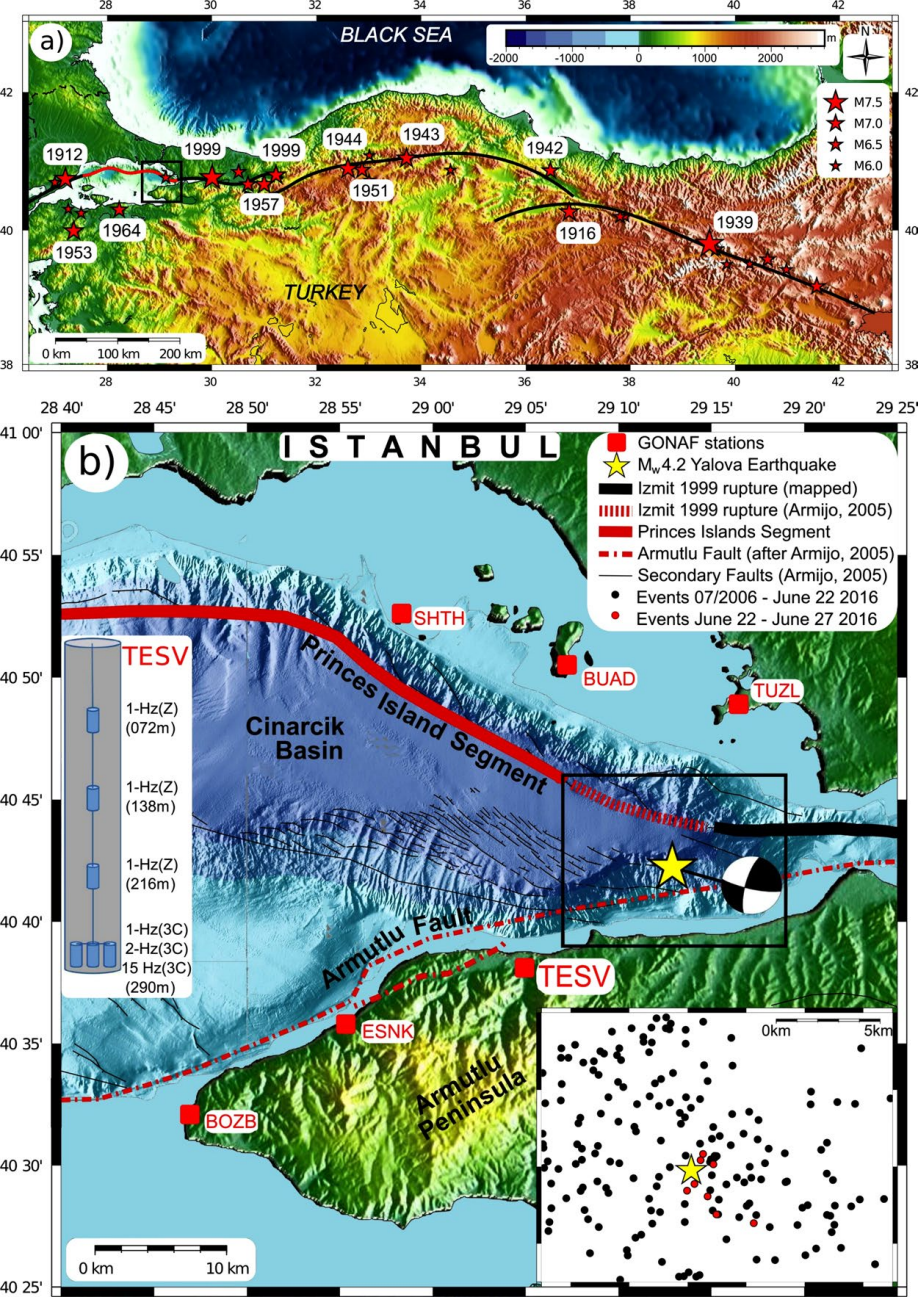
(**a**) The North Anatolian Fault system in Turkey. Location map of the North Anatolian Fault Zone (NAFZ) as the plate-bounding transform fault separating Eurasia from Anatolian^14,21^. Stars (M > 6) and years (M > 6.8) mark large earthquakes along the NAFZ since 1912, including the 1999 Izmit and Düzce events^28,40^. During the last century, the entire NAFZ was activated except for the Marmara section offshore Istanbul (indicated by the red line), where the last large (M > 7) earthquake occurred in 1766^16,24,25^. The focus area of the study is the eastern portion of the Marmara section of the NAFZ indicated by the black square (enlarged in **b**). This figure was created using GMT (Generic Mapping Tools) version 4.5 available at http://gmt.soest.hawaii.edu/projects/gmt/wiki/Download. (**b**) Eastern Marmara region, GONAF stations and location of the M_w_4.2 event. Location of the June 25, 2016, M_w_4.2 earthquake (yellow star) below the eastern part of the pull-apart Cinarcik Basin where the NAFZ branches into the Armutlu fault and the Princes Islands segment. Locations of GONAF borehole-geophone arrays are indicated in red^13^. The schematic sketch on the left shows a cross section of the TESV sensor distribution with four levels of 1, 2, and 15 Hz vertical and 3-component geophones. The black rectangle is enlarged in the lower right showing epicenters of seismicity during the last decade (black dots) and the eight strongest events (red dots) of the seismic sequence framing the M_w_4.2 mainshock. Epicenters are local seismicity from the preceding decade and determined from the permanent regional seismic network operated by the Turkish Disaster and Emergency Management Presidency of Turkey (AFAD)^31^. This figure was created using GMT (Generic Mapping Tools) version 4.5 available at http://gmt.soest.hawaii.edu/projects/gmt/wiki/Download.

The NAFZ separates the Anatolian and Eurasian plates, extending for 1200 km between the Karliova triple junction in eastern Anatolia and the Gulf of Saros, Northern Aegean Sea^14–16^ (Fig. 1a). The westward movement of Anatolia has developed in the tectonic framework of the northward moving Arabian plate^17,18^. It is connected to southward rollback of the Hellenic subduction zone, where the African lithosphere is subducted below the Aegean plate^19,20^. It has an average GPS slip rate of 20–25 mm/yr, increasing towards its western end^17^. A dominantly strike-slip fault zone along the bulk of the NAFZ turns into a more complex transtensional system of fault branches in NW Turkey^15,21^.

Starting in 1939, seven M~7 earthquakes occurred between 1939 to 1999 whose epicenters progressed sequentially westward along the NAFZ, arriving at the eastern end of the Sea of Marmara in 1999^15,22–25^ (Fig. 1a). The 1999 M_w_7.4 Izmit and M_w_7.1 Düzce mainshocks resulted in the death of >20.000 persons^26,27^. This left the Marmara section as the only segment that has not produced a large earthquake since 1766. The average recurrence rate on this section is around 200–250 years^16^. The cumulative moment release in the Marmara region has doubled since the 1999 events^25^. Nonetheless activity along the main fault branch below the Sea of Marmara is sparse. Few M > 4 earthquakes have occurred, and several aseismic fault patches were identified that could serve as nucleation points for the pending M > 7 event^25,28,29^.

The 5-day TESV-site equipment test captured the seismic activity surrounding the magnitude M_w_4.2 earthquake at 05:40:15.18 UTC on 25 June 2016. This includes at least 18 foreshocks that immediately preceded the M_w_4.2, the largest regional earthquake in several years.

To test for foreshocks of much lower magnitude than an M_w_4.2 earthquake, we need a corresponding earthquake-catalogue completeness magnitude M_c_. In this case, M_c_ should be ideally on the order of −1, preferably even lower. Burying seismometers 300 m underground can readily lower a networks detection threshold by as much as 2 or more magnitude levels, depending on local conditions. This type of installation results in an order of 5 to 50-fold increase in detection of small earthquakes^12,13,30^. Except for the SHTH site, which for our study period had only a surface station in operation, the data discussed here come from the multi-level arrays reaching this depth (see Fig. 1b). Especially important were the bottom 1 Hz and 2 Hz 3-C sensors, as these recorded clear S-waves, thereby determining hypocentral distances.

The sampling rate of our TESV data was set at 500 Hz. Except for a half hour gap starting at 12:30 UTC on 23^rd^ June 2016, the sensors of the TESV array recorded continuously from 08:00 UTC on 22^nd^ June to 08:00 UTC on 27^th^ June 2016 – a full 5-day span. The M_w_4.2 earthquake occurred in the middle of the TESV test data set, at 05:40:15.181 UTC on 25^th^ June 2016.

The TESV borehole array allowed us to record approximately 7 times the number of events in the M_w_4.2 sequence as the combined, surface-based, Turkish national networks. During the 5-day start-up test at least 61 more events with magnitudes ranging from M_w_ = 0 to M_w_ = 3.5 were detected at TESV as coming from the M_w_4.2 rupture area. Some of the larger events were also recorded at other GONAF borehole arrays in operation at that time (ESNK and BOZB) and a few of these were also recorded at the island-based SHTH surface sensor. Including the mainshock, a total of 9 events – 3 before the M_w_4.2 – were strong enough to be located by national seismic networks^21^ (red dots in the inset of Fig. 1b).

## Methods and Results

The 62 earthquakes were located in an area with diffuse background seismicity in the preceding decade^31^ (black dots in the inset of Fig. 1b). To compare the space-time relationships and waveforms of these events to the background activity, we applied both statistical and signal processing methods. Our statistical analysis compared inter event times and distance for the 62 events with all other events surrounding the epicenter as listed in the Turkish national catalogue of AFAD^31^. According to the ANOVA analysis of variance test^32^, their clustering is significantly different from the background seismicity in the same area during the rest of 2016.

**Figure 2:**
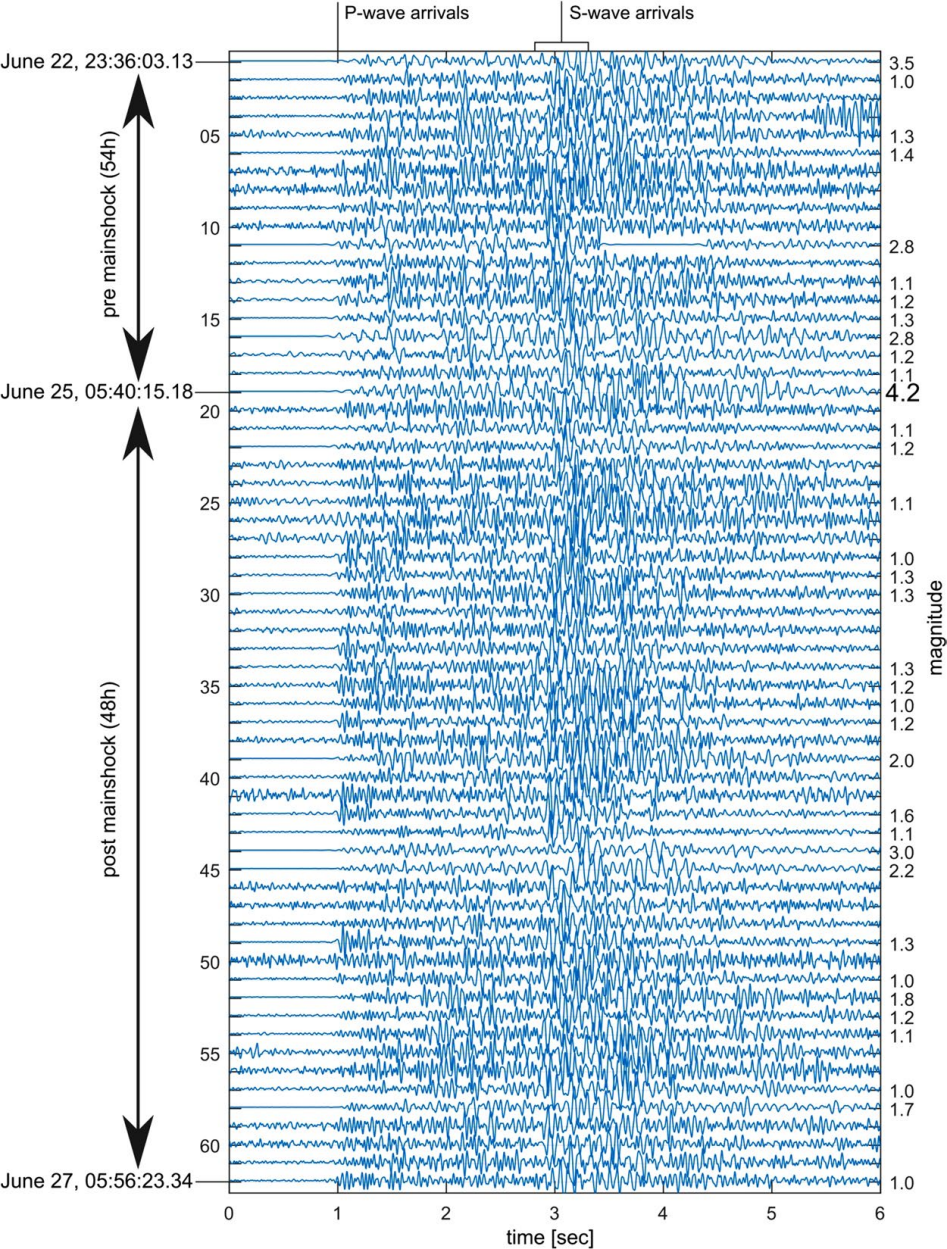
Vertical-component waveform recordings for the 62-event Yalova seismic sequence. Vertical-component waveform recordings of all 62 events recorded at the 1 Hz downhole seismometer of the GONAF-TESV array (see Table for details of the events). Amplitudes are normalized within each trace.

We filtered out electrical and low-frequency seismic noise from our seismograms by applying a fourth order Butterworth band pass filter between 3 and 45 Hz. We also inspected the spectrogram of these recordings as a function of sensor depth^13^. In this way, we visually identified 110 earthquakes in the five days of data. Of these, we found a total of 61 events with sufficiently high signal-to-noise ratio to accurately measure S-P differential arrival times. With these data it was possible to pick S-P times relative to that of the M_w_4.2 to within 0.1 s or less. The average S-P time of all 62 events is 2.00 s +/− 0.07 s. Their waveforms as recorded at the 290-m deep 1 Hz vertical seismometer of the TESV array are shown in Fig. 2.

**Figure 3:**
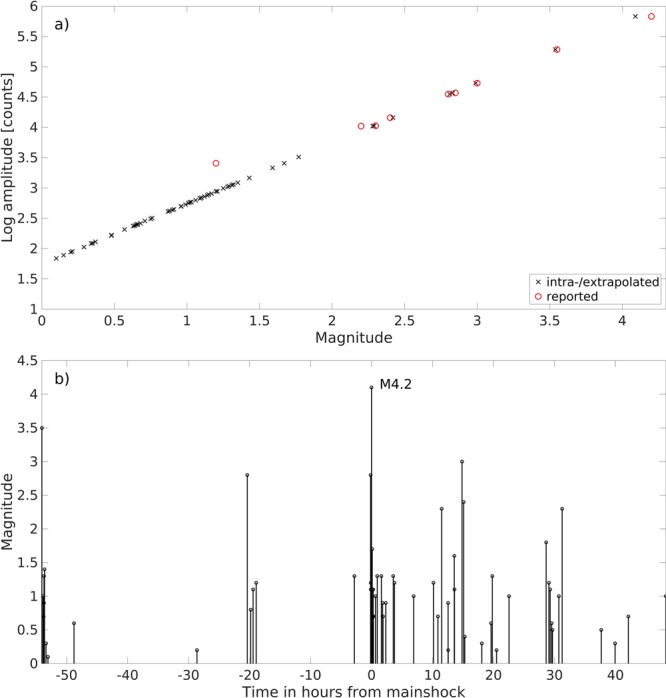
(**a**) Event magnitudes of the 62-event sequence. Linear regression magnitude determination for the 53 fore- and aftershocks of the M_w_4.2 event detected by the TESV borehole seismic array. For the nine largest events magnitudes were available based on surface recording of the regional permanent stations (red circles). Additional magnitudes were determined based on the extrapolating the linear regression and using amplitudes recorded at the GONAF-TESV vertical seismic array (black x). (**b**) Temporal occurrence of the event magnitudes for the 62-event sequence. Chronological plot of the magnitudes of the entire 62-event sequence in hours with respect to the mainshock.

To assign magnitudes for the 62 events, we compared TESV P-wave amplitudes to the nine events for which magnitudes were determined by the national network. These magnitudes ranged between 4.2 and 1.2. Aligning the nine reported magnitudes against their amplitudes recorded at the TESV, we determined the relation M = log(A_TESV_) − 1.74 and used it to estimate the magnitudes of the remaining events (Fig. 3a). This resulted in magnitudes for the entire sequence ranging from 0.1 to 4.2 (see Table 1). Plotting the magnitudes in chronological order shows a very rough trend toward increasing values leading up to the M_w_4.2 mainshock. This is followed by a typical aftershock sequence, with the largest aftershock being about one magnitude step (M ~ 3) smaller than the mainshock (Fig. 3b).

**Table 1:** Information on source times, S-P times and magnitudes for all 62 events.

Event no	P-wave arrival time (UTC)						S-P-time	Estimated magnitude
	Year	Month	Day	Hour	Min	Sec		
**1**	**2016**	**6**	**22**	**23**	**36**	**3.065**	**1.961**	**3.5**
2	2016	6	22	23	47	16.752	1.958	1
3	2016	6	22	23	53	34.322	1.993	0.7
4	2016	6	22	23	56	27.977	1.957	0.9
5	2016	6	22	23	56	32.34	1.928	1.3
6	2016	6	23	0	2	17.633	1.99	1.4
7	2016	6	23	0	17	43.453	1.97	0.3
8	2016	6	23	0	36	21.458	1.886	0.1
9	2016	6	23	4	52	46.351	1.924	0.6
10	2016	6	24	1	2	29.597	1.954	0.2
**11**	**2016**	**6**	**24**	**9**	**17**	**50.594**	**1.994**	**2.8**
12	2016	6	24	9	51	56.515	1.966	0.8
13	2016	6	24	10	14	53.767	1.936	1.1
14	2016	6	24	10	45	16.751	1.967	1.2
15	2,016	6	25	2	51	4.804	2.017	1.3
**16**	**2016**	**6**	**25**	**5**	**30**	**56.013**	**1.987**	**2.8**
17	2016	6	25	5	31	55.812	1.976	1.2
18	2016	6	25	5	32	11.932	2.03	1.1
**19**	**2016**	**6**	**25**	**5**	**40**	**15.123**	**2.082**	**4.2**
20	2016	6	25	5	44	19.241	2.018	0.5
21	2016	6	25	5	45	40.074	1.983	1.1
**22**	**2016**	**6**	**25**	**5**	**46**	**4.657**	**2.004**	**1.7**
23	2016	6	25	5	49	48.808	1.992	0.7
24	2016	6	25	5	50	33.658	2.11	0.7
25	2016	6	25	5	56	23.357	2.135	1.1
26	2016	6	25	5	57	34.426	2.096	0.7
27	2016	6	25	5	59	34.711	1.966	0.7
28	2016	6	25	6	19	20.935	2.006	1
29	2016	6	25	6	36	28.414	1.996	1.3
30	2016	6	25	7	16	45.571	2.014	1.3
31	2016	6	25	7	28	37.867	2.006	0.9
32	2016	6	25	7	33	7.838	2.002	0.7
33	2016	6	25	7	59	47.393	1.945	0.9
34	2016	6	25	9	13	31.954	2.014	1.3
35	2016	6	25	9	25	44.973	1.97	1.2
36	2016	6	25	12	34	41.336	1.99	1
37	2016	6	25	15	49	9.032	1.941	1.2
38	2016	6	25	16	33	43.693	1.976	0.7
**39**	**2016**	**6**	**25**	**17**	**10**	**12.637**	**2.073**	**2.3**
40	2016	6	25	18	13	15.467	2.148	0.9
41	2016	6	25	18	14	18.636	1.932	0.2
42	2016	6	25	19	13	55.63	1.938	1.6
43	2016	6	25	19	16	6.656	1.924	1.1
**44**	**2016**	**6**	**25**	**20**	**30**	**28.73**	**2.246**	**3**
**45**	**2016**	**6**	**25**	**20**	**46**	**42.12**	**2.204**	**2.4**
46	2016	6	25	20	58	15.772	1.996	0.4
47	2016	6	25	23	45	29.288	2.02	0.3
48	2016	6	26	1	17	5.058	2.063	0.6
49	2016	6	26	1	28	51.2	1.927	1.3
50	2016	6	26	2	10	58.412	2.021	0.2
51	2016	6	26	4	12	55.288	1.964	1
52	2016	6	26	10	18	33.212	1.977	1.8
53	2016	6	26	10	45	20.773	2.088	1.2
54	2016	6	26	10	58	32.066	1.921	1.1
55	2016	6	26	11	12	12.279	1.979	0.6
56	2016	6	26	11	19	45.253	1.95	0.5
57	2016	6	26	12	22	59.097	1.96	1
**58**	**2016**	**6**	**26**	**12**	**55**	**41.733**	**2.104**	**2.3**
59	2016	6	26	19	20	44.396	1.952	0.5
60	2016	6	26	21	36	21.5	1.94	0.3
61	2016	6	26	23	46	44.126	2.136	0.7
62	2016	6	27	5	56	23.339	2.05	1

Table with event no., source time, S-P differential arrival time and event magnitudes for the entire 62-event Yalova seismic sequence detected by the GONAF-TESV vertical seismic array.

**Figure 4:**
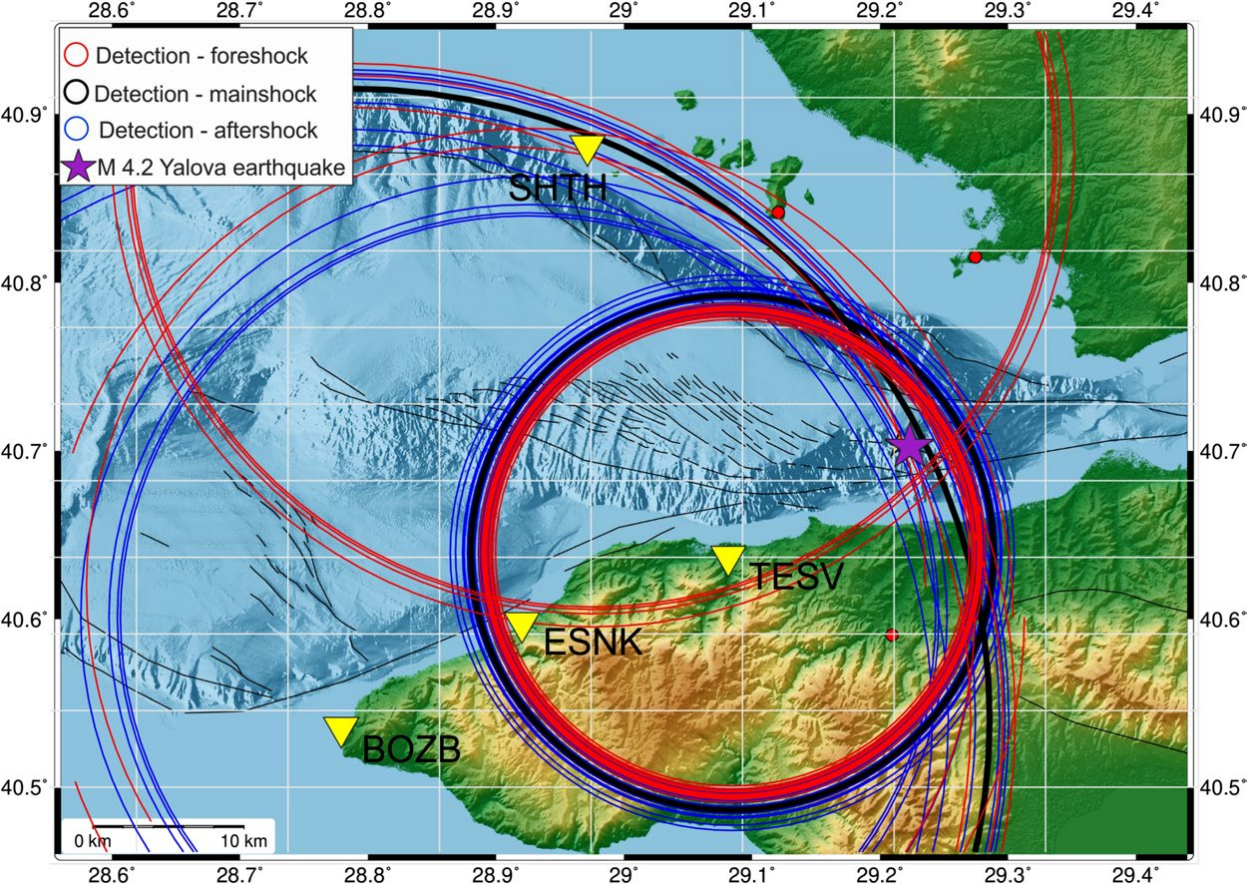
Estimated epicenter locations for events detected at 4 stations of the GONAF network. Estimated epicentral distances for events of the 62-event sequence framing the M_w_4.2 Yalova earthquake including data from borehole GONAF stations (BOZB, ESNK, TESV) and a surface station on the Princes Island Sivriada (SHTH). The purple star indicates the epicenter of the M_w_4.2 Yalova mainshock as reported by the Turkish national network operated by AFAD. Red and blue circles are results for fore- and aftershocks, respectively, while the black circles are results for the M_w_4.2 event. The results show that the S-P times for the same station across the different events are very consistent, and therefore, the location of these events is suggested to be on the fault patch activated by the mainshock. The precision of the distance as determined from the S-P differential arrival time is estimated to be on the order of 350 m. This figure was created using GMT (Generic Mapping Tools) version 4.5 available at http://gmt.soest.hawaii.edu/projects/gmt/wiki/Download.

We do not have enough recordings with good azimuthal coverage for accurately determining the hypocenters of each of the foreshocks. We were, however, able to estimate epicentral distances using S-P differential travel times recorded at the GONAF borehole stations BOZB, ESNK, and TESV. S-P data was also available from the SHTH surface station on the Princes Island Sivriada (Fig. 4). The epicentral circles are estimated to have a radial precision of about 350 m. Their intersections are concentrated around the epicenter associated with the mainshock. Based on the scaling relations of Bohnhoff *et al*.^33^ they cover a patch on the order of 1 km². The projection of these circles to the estimated hypocentral depth (11 km) of the M_w_4.2 further reduces the space containing the 61 additional events.

**Figure 5:**
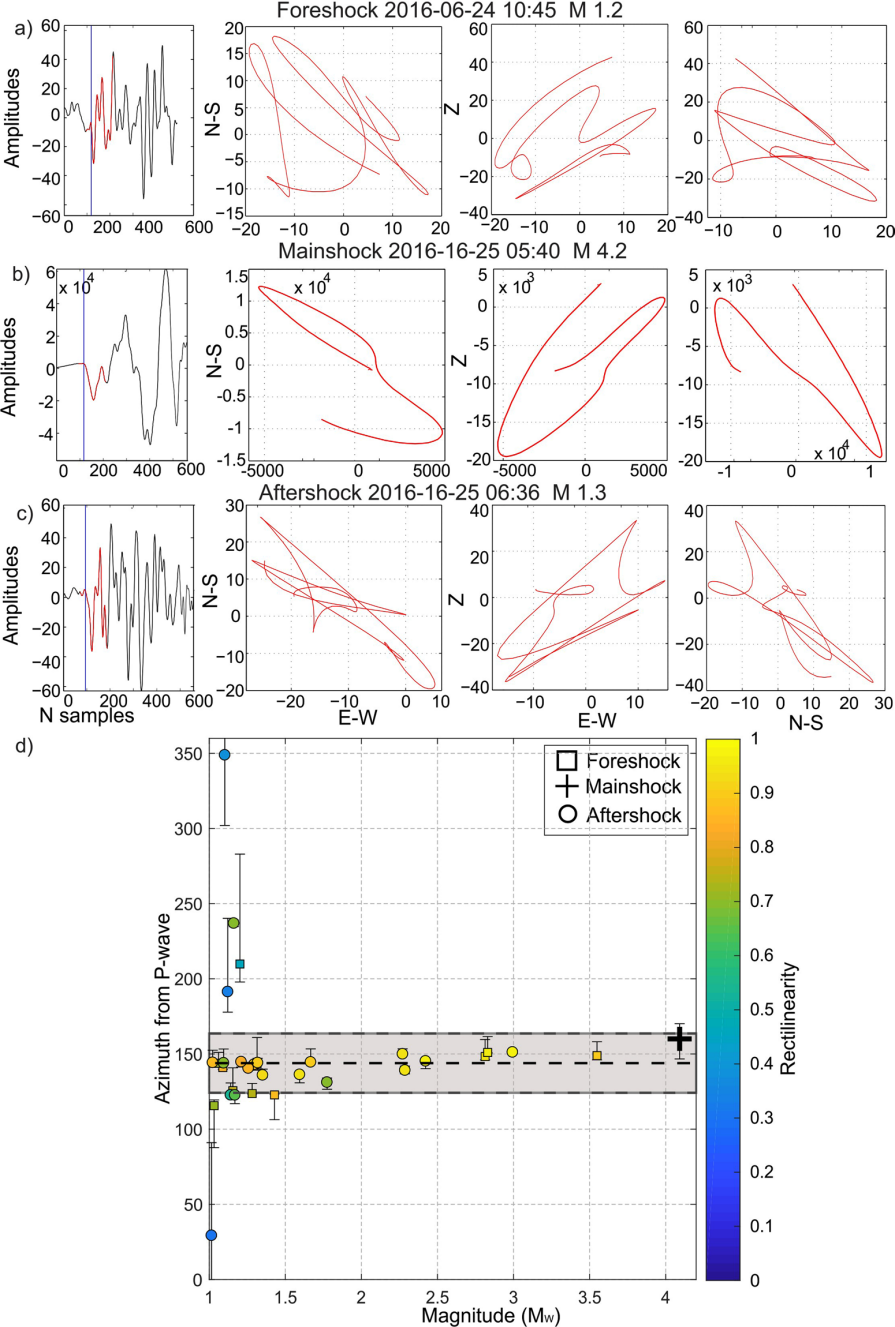
P-wave polarizations consistent with the back azimuth of the M4.2 event: Results from the particle motion and polarization analysis. (**a**–**c**) Particle motion plots recorded at the 2 Hz 3-C borehole geophone of the TESV array for 3 example events with magnitudes between 1.2 and 4.2. Shown from left to right are the vertical seismogram, the EW- depth, and the NS-depth particle motion, respectively. (**d**) Relative back azimuths for events with magnitudes M > 1 as determined from the polarization analysis based on particle motions. A consistent value of 145° for the back azimuth is obtained for the larger events while the scatter starts to increase for the smallest events due to reduced signal-to-noise ratios. Squares, crosses and circles indicate foreshock, mainshock and aftershocks, respectively. The color is encoded with the rectilinearity value obtained for each event. The grey rectangle frames one standard deviation of the azimuths obtained for the events with M > 1.3. The general indication is that due to the consistent back azimuth together with the uniform S-P differential travel time the entire 62-event sequence presented in this study could originate from the same fault patch that was activated during the M_w_4.2 mainshock.

To help constrain this source volume, a polarization analysis to the sequence’s P wave particle motions was done. For this study, the 2 Hz 3-C borehole sensors of the TESV array was used. Stable back-azimuths were found for M > 1 events (Fig. 5). The consistent back azimuth together with the uniform S-P differential travel times for the 62-events suggests that they all originated from the same 1–2 km long zone that failed during the M_w_4.2 mainshock.

**Figure 6:**
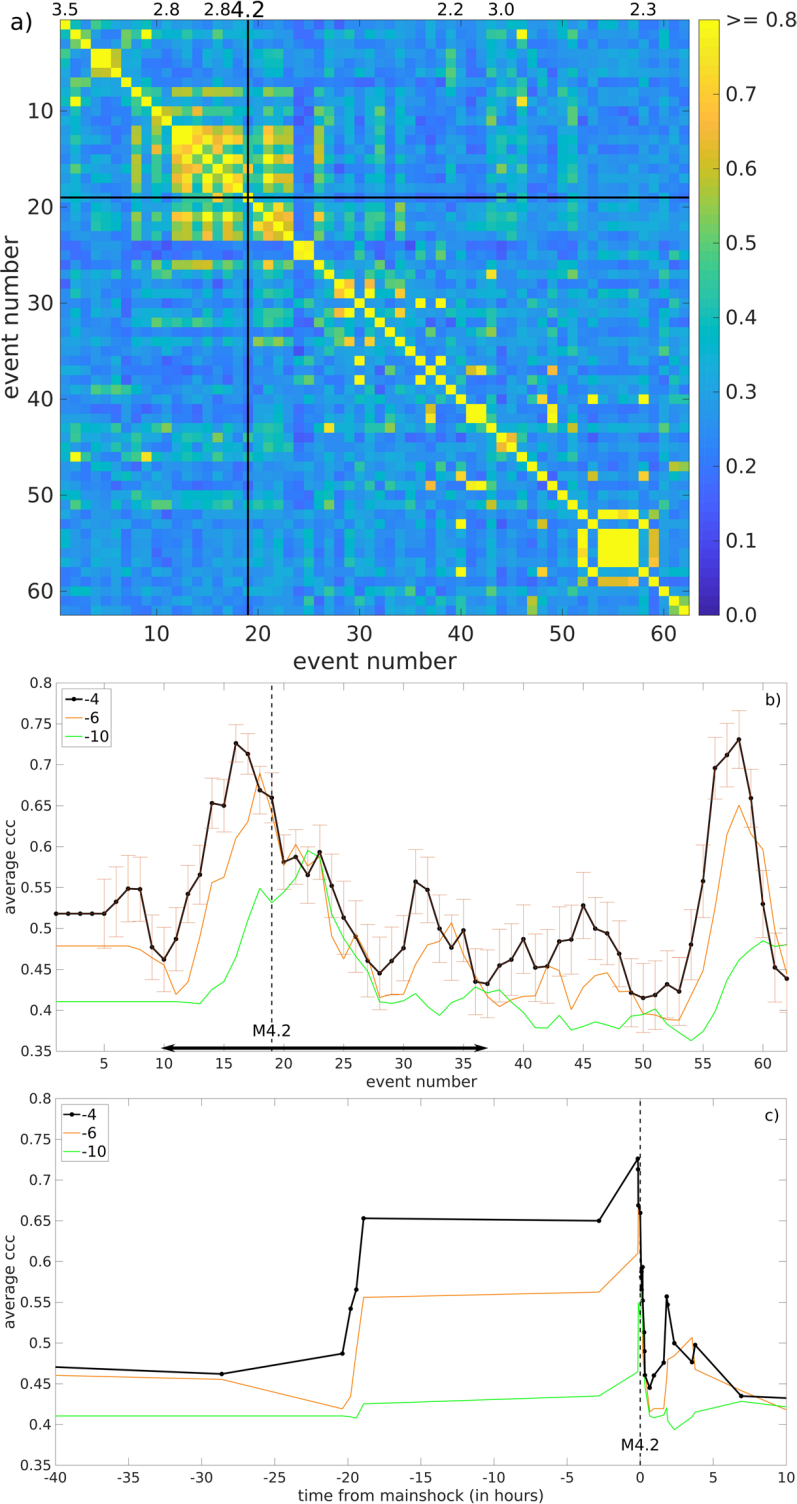
(**a**) Waveform cross-correlation matrix for the entire 62-event sequence. The color-coded waveform cross-correlation matrix of pairwise correlation of the 62-event sequence. The events are listed in chronological order along the left and bottom edges of the matrix. The matrix is symmetric about the diagonal, along which their autocorrelations lie. The correlation color scale is cut off at 0.8 in order to better display the variability in the coefficients. The M_w_4.2 mainshock is shown by the black line. (**b**) Running average cross-correlation coefficients for the entire 62-event sequence. Running average cross-correlation coefficients (ccc) plotted chronologically equidistantly with event numbers for window lengths including the past 5 (black), 7 (orange), and 11 (green) events showing a clear increase and maximum prior to the M_w_4.2 mainshock. Vertical blue error bars point out the standard error of the mean at each calculated average value. The horizontal black bar indicates the time window plotted in (**c**). (**c**) Running average cross-correlation coefficients plotted in true time. Same as in (**b**) but plotted in true time for the time window 40 hours prior to 10 hours after the M_w_4.2 mainshock indicating an elevated ccc plateau in the 20 hours preceding the mainshock. The dashed vertical line indicates the time of the M_w_4.2 mainshock.

To quantify their similarity, we cross-correlated event waveform pairs as recorded on TESV’s 290 m deep 1 Hz vertical sensor. We de-trended and tapered the waveforms in 4.1 s long windows, starting 0.1 sec prior to the P-wave onset. These windows thus included both the P- and S-wave arrivals and their codas. Their cross-correlation maxima were arranged in a time sequential, square, 62 × 62-element, matrix with their autocorrelations lying along its diagonal axis^34^. The values of the resulting 1,891 coefficients range between 0.08 and 0.92 (Fig. 6a). The resulting matrix contains a high-correlation sub-matrix of events just before the M_w_4.2, and a less extensive one during the aftershocks. Running averages of the coefficients with varying window lengths shows their time-dependent trends. The averages were calculated in a retrospective manner: the results include only events prior to the time point shown (Fig. 6b). These averages increased about 20 hours prior to the mainshock, reaching a maximum 10 minutes before the 4.2 mainshock (Fig. 6c). The results also illustrate how our correlation method might be implemented in an earthquake forecasting system involving real-time signal processing and waveform cross-correlation.

**Figure 7:**
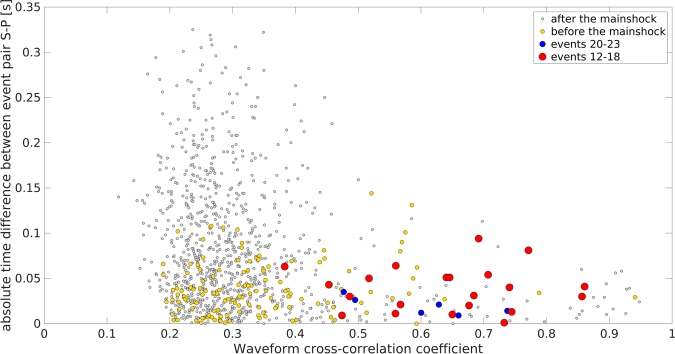
Cross-correlation coefficients for event pairs plotted with differential S-P time. Cross-correlation coefficients for all event pairs plotted with their difference in S-P time. Orange and red dots show event pairs for early (events 1–11) and immediate (events 12–18) foreshocks. Blue and gray dots show event pairs for immediate (events 20–23) and later (from event 24) aftershocks. The event pairs with the highest cross-correlation coefficients also have the smallest S-P time differences indicating that they could origin from the same fault patch activated by the mainshock. This is interpreted to reflect the emergent failure process leading to the M_w_4.2 mainshock.

The events at the beginning of the sequence and those towards the end show larger differences in S-P times and smaller cross-correlation coefficients (Fig. 7). This indicates larger spatial differences in their hypocentral location. In contrast, the events surrounding the mainshock show the smallest S-P differences and highest cross-correlations, suggesting their close spatial relationship with the M_w_4.2 earthquake.

## Conclusions

We report on the borehole-based detection of a 62-event sequence framing an M_w_4.2 mainshock along the Marmara seismic gap offshore of Istanbul in NW Turkey. The similarity of the earthquakes reported here indicates that they occurred within several hundred meters of the mainshock – in other words within its estimated source area^33^. We found three lines of evidence supporting this conclusion. First, the epicentral circles determined by S-P times at four GONAF sites intersect within a few hundred meters of the M_w_4.2 location. Second, the P- wave particle motions of best resolved M > 1 events consistently point to the same back azimuth as the M_w_4.2. Third, the waveforms of these events are more strongly correlated in time and space than other events in the 1^st^ and 2^nd^ halves of 2016.

In part we observe a set of foreshocks with increasing waveform similarity during the hours before the M_w_4.2 earthquake that show a similar behavior as foreshocks repeatedly observed during laboratory rock deformation test and more recently before large plate-bounding earthquakes.

The exact locations of the events discussed here could not be fully constrained since their magnitudes were too small for them to be registered at the regional surface-networks or the other operational GONAF stations. The lack of even more sensitive or nearer stations precluded testing, for example, the inverse Omori law for foreshocks^35^, where the rate of earthquakes before a mainshock increases according to a power law. The same applies for both (1) a decrease in b-values, as posited for near-offset events preceding a mainshock and (2) for the migration of foreshocks toward the mainshock.

Well-documented field evidence for foreshock behavior in nature is still sparse. Our example is one of only a few field-based observations of much smaller, near-hypocenter seismicity preceding a mainshock. Consequently, there are a number of explanations for the relationship of foreshock to mainshocks. These include, for example, the controversial cascade model where earthquakes trigger aftershocks larger than themselves^5,36^. Foreshock laws are partly still seen as statistical in nature, observable when averaging over a large number of sequences, but not systematically for every event^35^.

In this light, the progress in monitoring instrumentation and its operation in a seismically active region that is discussed here may hold promise for hazard and risk reduction. Going underground with seismic monitoring, as it were, can work even in highly urbanized areas. Adding to this technology the sort of analysis methods we present here can contribute towards refining operational earthquake forecasting, even perhaps helping plan activities such as critical evacuations^37–39^.
